# Stereological reconstructions of 3D cellular microstructures by combining adversarial learning and Voronoi tessellations

**DOI:** 10.1038/s41598-026-52851-7

**Published:** 2026-05-13

**Authors:** Lukas Fuchs, Thomas Wilhelm, Orkun Furat, Volker Schmidt

**Affiliations:** 1 Institute of Stochastics, Ulm University, Ulm, 89069 Germany; 2 SDU Applied AI and Data Science Unit, University of Southern Denmark, Odense, 5230 Denmark

**Keywords:** Stereology, Cellular structure, Generative adversarial network, Spatial statistics, Tessellation, Microstructure modeling, Engineering, Materials science, Mathematics and computing

## Abstract

A novel stereological framework to generate synthetic three-dimensional cellular material structures using Voronoi tessellations is presented. While conventional investigations of microstructural features rely on costly and often destructive three-dimensional imaging techniques, our method enables the reconstruction of 3D cellular structures from two-dimensional planar-sectional image data. By representing 3D cell architectures through Voronoi tessellations, we obtain an analytical representation requiring only three parameters per cell, ensuring efficient storage and computational processing. Our framework employs a differentiable approximation of Voronoi tessellations combined with a discriminator neural network in an adversarial learning context, enabling gradient-based optimization of tessellation parameters to generate random 3D cellular structures with statistically similar 2D planar sections as observed in measured 2D image data. We demonstrate the framework on image data of various cellular materials including metallic alloys, biological cells, and foam structures. The presented framework shows state-of-the-art capability of stereologically reconstructing 3D cellular microstructures, while introducing a low-parameter representation, preserving physical interpretability, and ensuring computational efficiency.

## Introduction

The effective macroscale properties of materials, including mechanical strength, electrical conductivity, thermal conductivity, and hardness, are fundamentally influenced by their underlying microstructure^1–3^. These structure-property relationships have been extensively documented for various material systems, from metals and ceramics to biological tissues and composite materials^4–6^. Recent advances in experimental techniques and computational methods have further emphasized the critical role of microstructure in determining the behavior of the material under various conditions^7,8^, establishing microstructural design as a key part of modern materials science and engineering^9,10^.

Many natural materials exhibit a so-called cellular microstructure, in which a material is composed of individual cells or domains with distinct properties despite sharing the same chemical composition^11^. This cellular microstructure is, for example, observable in metallic materials where cells correspond to regions showing the same orientation of the crystal lattice^12–14^, in biological systems such as the cellular structures of plants where cells correspond to plant tissue compartments^15,16^, synthetic materials including foams where cells correspond to gas-filled pores enclosed by a solid matrix^17,18^, or rocks where cells correspond to mineral grains^19^. The spatial arrangement, size distribution, the boundary between individual cells and their morphological features critically determine the macroscopic effective properties of the bulk material^20,21^.

Despite the importance of the three-dimensional (3D) cellular structure of materials, comprehensive investigation of these microstructures often presents significant challenges due to the limitations of 3D imaging techniques^22,23^. For example, focused-ion beam scanning electron microscopy (FIB-SEM) provides high-resolution data. However, these imaging techniques are inherently destructive, requiring complete sectioning of the specimen^24^. Even typically “non-destructive” measurement techniques such as X-ray tomography can induce damage in sensitive materials through radiation exposure^25^. Furthermore, in various materials, the cellular microstructure is discernible on a length scale for which 3D imaging is often prohibitively expensive in time and cost^26–28^.

However, 3D microstructural data remains essential for many applications, including accurate numerical simulations of material behavior^29,30^, quantitative spatial analysis of phase distributions^3,31^, and the development of structure-property relationships^6^. Simulations based on two-dimensional (2D) data often fail to capture the full complexity of a material’s structure, particularly for phenomena such as crack propagation, phase transformations, and deformation mechanisms, which are inherently three-dimensional^10,30^. Such applications drive the need for methods that can reliably generate representative 3D microstructures from more accessible 2D image data.

Consequently, there is significant interest in developing algorithms that (re-)construct 3D microstructures from 2D image data. As a result, several stereological approaches have been proposed in the literature. These include statistical reconstruction methods^32–34^, where correlation functions derived from 2D images guide the generation of virtual 3D structures. Machine learning approaches, particularly those based on generative adversarial networks (GANs)^35^, have emerged as powerful tools for this task^36–42^. More recently, diffusion models have been employed to generate large-scale 3D images of microstructures from a single 2D image^43,44^. Descriptor-based methods have also been adapted to reconstruct virtual 3D microstructures such that those match given descriptor statistics^45^ as well as multiple point statistics-based methods^46,47^. While demonstrating considerable success, these mostly voxel-based approaches typically require extensive parameterization, leading to outputs that lack direct physical interpretability. Furthermore, often stereological methods are limited to generating two- or three-phase grayscale images, making them challenging to apply to virtual cellular structure generation.

Tessellations, in particular distance-based tessellations such as Voronoi or Laguerre tessellations, have been shown to be a powerful tool for representing cellular microstructures, as they naturally partition space into convex polyhedra, often also referred to as cells^48^, allowing a low parametric and continuous representation of complex microstructures^14,49^. For instance, Voronoi tessellations are utilized to define an implicit geometric representation for multiphase interface tracking^50^. Laguerre tessellations obtained via partial optimal transport enable the construction of constant-volume Lagrangian meshes with free boundaries^51^ and can be used as the basis for structures in optimal transport–based Lagrangian shape optimization approaches^52^. In addition, there is a large interest in fitting tessellations to image data^53–55^ to generate virtual cellular structures^56,57^, or using them for the simplification of simulations^58,59^.

In this paper, we present a novel stereological framework capable of generating 3D cellular microstructures using random Voronoi tessellations^60,61^ that will be calibrated to image data depicting planar sections of cellular structures. The resulting analytical 3D representation offers several advantages: it can be transformed into a voxel grid at any desired resolution without introducing interpolation errors; it inherently produces convex cells with planar facets that are beneficial in numerical applications and meshing; furthermore, the presented stereological framework facilitates a low parametric representation of 3D cellular microstructures, that is, three parameters per cell, and allows direct interpretability of cell size distributions and spatial arrangements of cells^62,63^. The presented framework shows a structure similar to GANs, incorporating a discriminator neural network that distinguishes between planar sections of artificially generated microstructures and measured ones, enabling gradient descent-based optimization of the underlying tessellation to generate 3D microstructures with statistically similar 2D planar sections as observed in measured 2D data.

By bridging the gap between accessible 2D imaging and the need for 3D structural data, our method enables more efficient investigation of structure-property relationships in cellular materials without requiring destructive or costly 3D imaging techniques. The framework maintains physical interpretability, implicitly resolves measurement artifacts, and allows for enormous dimension reduction while offering flexibility for adaptation to various material systems.

## Methods

This section introduces a computational framework for constructing 3D cellular microstructures from 2D image data using differentiable Voronoi tessellations in an adversarial learning context. The methods address the fundamental challenge in materials characterization: obtaining representative 3D microstructural information from more accessible 2D image data. The framework consists of four main components: (1) a 3D differentiable approximation of a tessellation for cellular microstructure generation, (2) a slicing operation that extracts 2D planar sections from these 3D approximations, (3) a discriminator network that evaluates how well these generated planar sections match 2D image data of real cellular structures, and (4) a GAN-like gradient descent-based optimization procedure for the generation of realistic 3D cellular structures.

### Cellular structure and RGB representation

In the present paper, a cellular structure is defined as a collection of $n>0$ cells within a bounded observation window $W\subset \mathbb {R}^3$. Mathematically speaking, the cellular structure can be represented by a function $C:W\rightarrow [0,1]^n$, defined as

1 $$\begin{aligned} C(x)=(C_1(x),C_2(x),\dots ,C_n(x)) \quad \text {for each } x\in W, \end{aligned}$$

where $C_i(x) \in [0,1]$ denotes the likelihood that the point $x\in W$ belongs to the *i*th cell. The equality $C_i(x)=1$ indicates that *x* certainly belongs to cell *i*, while $C_i(x)=0$ indicates that *x* certainly does not belong to cell *i*. Furthermore, it is assumed that $\sum _{i=1}^n C_i(x) > 0$ for all $x \in W$, which means that each point of the observation window has a positive likelihood of belonging to at least one cell. In segmented image data, $C_i(x)$ takes only the values 0 or 1. For simplicity, in the present work the observation window *W* is assumed to be cubic, in particular $W = [0,L] ^3$ for some $L \in (0,\infty )$. Note that imaging techniques typically observe cellular structures only on a finite set of points, that is, it is not possible to determine the values *C*(*x*) of the function given in Eq. (1) for all $x\in W$. Thus, in applications, the value *C*(*x*) is typically only known for $x\in G$ from a discrete and finite pixel or voxel grid $G \subset W$.

The cellular structure representation *C* on a discretized grid *G* forms a large tensor of shape $|G| \times n$, where |*G*| denotes the number of grid points (pixels in 2D or voxels in 3D) and *n* is the number of cells. Such high-dimensional data is difficult to visually assess and, for large *n*, can result in substantial memory usage and computational overhead. To mitigate these issues, we introduce an RGB-based embedding. Generally speaking, a unique RGB color is assigned to each cell, and the resulting color map is interpreted as an RGB image. This reduces the dimensionality by a factor of approximately $n/3$. More precisely, the *i*th cell of a cellular structure in the observation window *W* with representation *C* given by Eq. (1) is assigned an RGB color $c_i \in [0,1]^3$ for each $i \in \{1,\ldots ,n\}$. Then, for each $x\in W$ the value *I*(*C*, *c*, *x*) of the RGB embedding is given by

2 $$\begin{aligned} I(C,c,x) = \sum _{i=1}^n c_i \cdot C_i(x), \end{aligned}$$

where $c=(c_1,\ldots ,c_n)$. For brevity, we use the notation *I*(*C*, *c*, *G*) to indicate that *I*(*C*, *c*, *x*) is evaluated for all $x\in G$ of some grid *G*. Furthermore, the nomenclature RGB embedding is also referred to as an RGB image.

### Voronoi tessellation

To represent 3D cellular microstructures observed in image data, distance-based tessellation models are commonly employed^64^, see also Ref.^53^. In the present study, Voronoi tessellations are specifically utilized. A Voronoi tessellation, denoted by $\mathcal {T}=\{T_i:i\in \{1,2,\dots ,n\} \}$, partitions the observation window $W=[0,L]^3$ parametrically into $n>0$ convex sets $T_1,T_2\dots ,T_n\subset W$, which naturally can be interpreted as the cells of a cellular microstructure. Formally, for a point pattern $S=\{s_1, \ldots , s_n\}\subset W$ of so-called seed points, which are considered to be the parameters of the Voronoi tessellation, the cell $T_i$ corresponding to the seed $s_i\in W$ for each $i\in \{1,2\dots ,n\}$ is given by

3 $$\begin{aligned} T_i = \{x \in W : d(x, s_i) \le d(x, s_j) \text { for all } j \ne i,~ j \in \{1,2,\ldots ,n\}\}, \end{aligned}$$

where $d(x, s)=\Vert x-s\Vert$ denotes the distance function induced by the Euclidean norm $\Vert \cdot \Vert$ in $\mathbb {R}^3$. Intuitively speaking, each cell $T_i$ contains all points $x\in W$ that are closer to $s_i$ than to all other seed points.

*Periodic boundary conditions*

In practical applications, the size *L* of the observation window $W=[0,L]^3$ is restricted to a value not much larger than the size of an average cell due to computational limitations. As a consequence, a substantial number of cells intersect with the boundary $\partial W$ of *W*, that is, $T_i \cap \partial W \ne \emptyset$ for some $i\in \{1,2,\dots ,n\}$. This introduces boundary effects, which complicate the accurate computation of geometric descriptors and lead to artifacts in numerical simulations^65^. To mitigate these boundary effects, tessellations with periodic boundary conditions are considered. More precisely, a cell $T_i$ of a Voronoi tessellation with periodic boundary conditions is given by Eq. (3), where the distance function *d* is replaced by a periodic function $\widetilde{d}:\mathbb {R}^3\times \mathbb {R}^3 \rightarrow [0,\infty )$, defined as

4 $$\begin{aligned} \widetilde{d}(x, y) = \min _{v \in \{-L,0,L\}^3} \Vert x - (y + v)\Vert \quad \text {for all } x,y\in W. \end{aligned}$$

Note that $\widetilde{d}$ ensures the continuation of the tessellation cells across the boundaries of *W*, thus mitigating edge effects. In the following, only Voronoi tessellations employing the modified distance function $\widetilde{d}$ in place of the distance function *d* are considered.

*Differentiable relaxation*

A Voronoi tessellation directly provides a parametric representation of a cellular structure by means of Eq. (1). More precisely, such a cellular structure is given by the function $C^S:W\rightarrow [0,1]^{n}$, where $C^S(x)=(C^S_1(x), C^S_2(x),\dots , C^S_n(x))$ for each $x\in W$ and the values of $C^S_i(x)$ are given by

5 $$\begin{aligned} C^S_i(x) = {\left\{ \begin{array}{ll} 1,\qquad & \text {if } x\in T_i\\ 0, \qquad & \text {otherwise}, \end{array}\right. } \end{aligned}$$

for each $i\in \{1,2,\dots ,n\}$ However, note that $C^S(x)$ does not provide any information on how to adjust the set of seed points *S* of a tessellation to ensure that $x \in W$ belongs to a specific cell or not. Consequently, in its current form, this representation prevents efficient implementation of the objective of the present paper, i.e. the optimization of the set of seed points *S* to produce a tessellation which statistically reflects measured cellular structures. To address this, a so-called differentiable relaxation of the tessellation representation given in Eq. (5) is utilized, using a softmax function over negative distances. More precisely, the values of $C^S_i(x)$ considered in Eq. (5) are replaced by

6 $$\begin{aligned} C^S_i(x) = \frac{\exp (-\beta \, \widetilde{d}(x,s_i))}{\sum _{j=1}^n \exp (-\beta \, \widetilde{d}(x,s_j))}, \end{aligned}$$

for all $x\in W$ and $i\in \{1,2,\dots ,n\}$, where $\beta \in (0,\infty )$ is a so-called temperature parameter. This differentiable relaxation of Voronoi indicator functions is closely related to the partition functions introduced in Ref.^66^. Note that by determining the index $i\in \{1,\ldots ,n\}$, which maximizes $C^S_i(x)$, the binary assignment of *x* to a specific cell, see Eq. (5), can be derived. This allows us to maintain the interpretability of the tessellation while enabling efficient optimization of the seed-point pattern. More precisely, for each tessellation cell, the function $C^S_i$ is differentiable with respect to each seed point $s\in S=\{s_1,s_2,\dots ,s_n\}$, which enables gradient-based optimization. This allows for the optimization of a loss function that quantifies the dissimilarity between a desired cellular structure and the tessellation induced by *S*. The temperature parameter $\beta$ influences the strength of relaxation in Eq. (6). For large values of $\beta$, the softmax function approximates a hard assignment, that is, the values $C^S_i(x)$ are close to zero or one for each $x\in W$, and thus the cell to which *x* belongs can easily be derived. In contrast, small values of $\beta$ lead to smoother transitions between cells and help prevent vanishing gradients during optimization. In this study, the temperature parameter is set to $\beta = 10$.

*RGB representation*

**Fig. 1:**
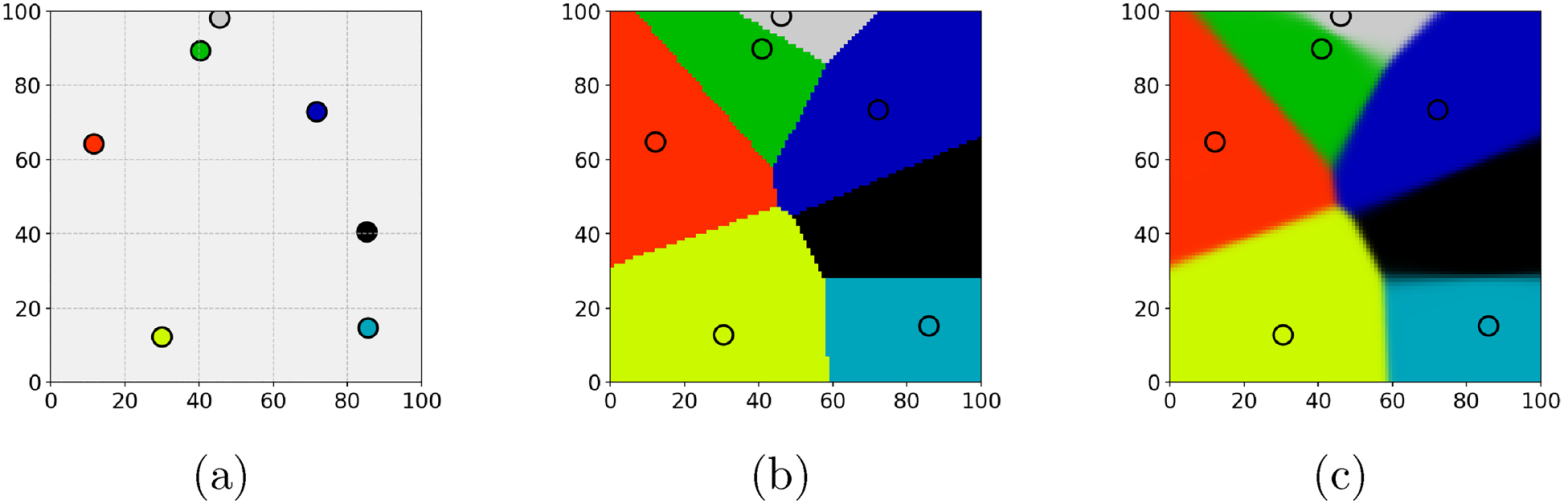
Schematic illustration of the differentiable RBG representation of a Voronoi tessellation. (**a**) Seed points in 2D space with corresponding unique RGB colors. (**b**) Corresponding Voronoi tessellation with cells given by Eq. (5), which exhibit sharp cell boundaries. (**c**) Corresponding softmax-based differentiable RGB representation of a Voronoi tessellation with smooth transitions between neighboring cells, where the cells are given by Eq. (6).

Note that the soft-max-based representation $C^S = (C_1^S,C_2^S,\dots ,C_n^S)$ of a Voronoi tessellation given in Eq. (6), generated by a set of seed points $S=\{s_1,\ldots ,s_n\}$, provides a cellular structure by means of Eq. (1). Furthermore, by assigning each cell a color $c^S_i\in [0,1]^3$, a 3D RGB encoding $I(C^S,c^S,x)\in [0,1]^3,~x\in W$, can be achieved using Eq. (2), where $c^S = (c^S_1,c^S_2,\dots ,c^S_n)$. Using an implicit kernel operations package such as the python package PyKeOps^67^, the RGB image computation can be performed memory efficient. A visualization of an RGB representation for a 2D Voronoi tessellation generated from a 2D point pattern is provided in Fig. 1. In Fig. 1a, the seed points are shown, each colored according to its assigned RGB value. When the cellular structure is represented using the definition of $C^S_i$ in Eq. (5), the resulting tessellation exhibits sharp cell boundaries, as seen in Fig. 1b. In contrast, applying the soft assignment formulation by means of Eq. (6) produces an RGB representation with smooth transitions between neighboring cells, see Fig. 1c. This enables differentiation of the components in the RGB vector $I(C^S,c^S,x)\in [0,1]^3$ with respect to the underlying seed point pattern *S*.

### Stereological framework for generating 3D microstructures

This section outlines the stereological framework used for generating 3D microstructures. The method takes measured cellular structures *C* on a grid *G* in the sense of Eq. (1) as input and produces 3D tessellations $C^S$ on a continuous window *W* in the sense of Eq. (5) as output. To enable a comparison between the grid-based input and continuous output, the 3D tessellation $C^S$ is first evaluated on a 2D pixel grid, and subsequently, the RGB embeddings of *C* and $C^S$ are compared by a discriminator. Finally, the set of parameters *S* is optimized in order to increase the statistical similarity between *C* and $C^S$.

*Planar sectioning of 3D Voronoi tessellations*

To statistically compare a continuous 3D tessellation within the observation window $W = [0, L]^3$ with a 2D cellular input structure whose values are only known for discrete points on a 2D grid, the continuous 3D tessellation is evaluated on similar 2D grids. For this, assume that $L\in \mathbb {N}=\{1,2,\ldots \}$ is a sufficiently large integer and let $G_{d,k} \subset \{0, \ldots , L - 1\}^3$ denote a planar grid orthogonal to the *d*-th coordinate axis at position $k \in \{0, \ldots , L - 1\}$, where $d \in \{1,2,3\}$ corresponds to the x-, y-, and z-directions, respectively. More precisely, $G_{d,k} \subset W$ is defined as

7 $$\begin{aligned} G_{d,k} = \left\{ (\ell _1, \ell _2, \ell _3) \in \{0, \ldots , L - 1\}^3 :\ell _d = k \right\} . \end{aligned}$$

For example, $G_{2,3} = \{0, \ldots , L - 1\} \times \{3\} \times \{0, \ldots , L - 1\}$ defines a planar 2D grid orthogonal to the y-axis at y-position $\ell _2=3$. By evaluating the RGB embedding of a tessellation $C^S$ using Eq. (2) at each point $\ell =(\ell _1,\ell _2,\ell _3) \in G_{d,k}$ for a pair $(d,k)\in \{1,2,3\}\times \{0,\dots ,L-1\}$, a 2D RGB image is generated. This image is comparable to the RGB embedding *I*(*C*, *c*, *G*) of some cellular structure *C* that was measured on some 2D grid *G*, making this representation suitable for statistical comparison of *C* and $C^S$, as described below.

*Discriminator architecture* Following the GAN approach from^35^, a discriminator network is trained to distinguish between the RGB image *I*(*C*, *c*, *G*) of a cellular structure *C*, derived from 2D measurements, and an RGB image $I(C^S,c^S,G_{d,k})$ derived from a Voronoi tessellation $C^S$ as outlined in the previous paragraph. Roughly speaking, the discriminator is trained to assign values close to one to RGB images originating from 2D input data of cellular structures and values close to zero to those originating from the tessellation. As a consequence, the discriminator implicitly learns key differences in the statistics of geometrical descriptors computed from the measured cellular structure and the tessellation, respectively^68^. By optimizing the set *S* of seed points of a tessellation such that the discriminator output for synthetic data approaches one, the statistical similarity between the input and generated microstructures increases. This adversarial training process allows us to iteratively refine the tessellation parameters to produce realistic 3D structures without explicitly defining similarity metrics. In particular, the optimization does not require any hand-crafted geometric descriptors or explicitly specified similarity measures, since the relevant features are learned implicitly by the discriminator during training.

Since for the generation of realistic cellular structures, local geometrical features are more critical than global ones, the architecture of the discriminator network is chosen to be fully convolutional. This class of discriminator architectures, often referred to as patch-based, has been shown to be effective for texture and microstructure classification, where local patterns are more relevant than global arrangements^69–71^.

As a consequence of the fully convolutional architecture, the discriminator $D:[0,1]^{h\times w} \rightarrow [0,1]^{h'\times w'}$ produces, for an RGB input image $J\in [0,1]^{h \times w}$ with height *h* and width *w*, an output image $D(J) \in [0,1]^{h' \times w'}$, where $h,h',w,w'\in \mathbb {N}$ are some integers such that $h> h' > 0$ and $w> w' > 0$. Each entry in *D*(*J*) represents the realism score of a small patch of *J*.

**Fig. 2:**
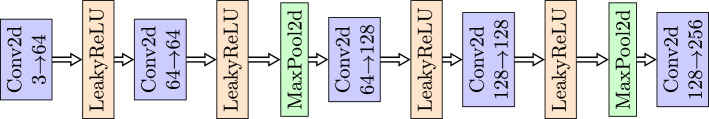
Overview of network architecture of the discriminator, where $n_\textrm{in}\rightarrow n_\textrm{out}$ denotes the numbers of input and output features $n_\textrm{in}$ and $n_\textrm{out}$ of the respective convolutional layers.

The architecture of the discriminator considered in this paper is based on a so-called VGG architecture^72^, with several key modifications to adapt it to the present application. Starting with a standard VGG19 network, the architecture is modified in the following way: First, the padding in all convolutional layers is removed. If the discriminator *D* is applied to periodic data (in particular, for generated periodic RGB images), periodic padding is applied instead. For non-periodic input data (in particular, for RGB images derived from experimentally measured data), no padding at all is applied. This ensures that boundary effects are handled consistently. Second, the ReLU activation function is replaced by a LeakyReLU function, where $\textrm{LeakyReLU}(x)=\max (\alpha x,x)$ for each $x\in \mathbb {R}$ with a slope parameter of $\alpha =0.2$, improving gradient flow^73^. Note that the LeakyReLU function is almost everywhere differentiable. Third, spectral normalization^74,75^ is applied to all convolutional layers. This normalization ensures that the gradients of the discriminator output with respect to *S* remain at an appropriate scale throughout training, preventing gradient explosion or vanishing gradient problems. Finally, the network is truncated to the first 11 layers to maintain an appropriate field of view ($24\times 24$ pixels) of the network, as deeper layers focus on more global features that may be less relevant for texture evaluation. A sigmoid activation function^76^ is applied to the final output layer to produce values in the range [0, 1]. The discriminator architecture is illustrated in Fig. 2 and more details of each layer are provided in the Appendix.

*Tessellation optimization* To generate realistic 3D microstructures, an adversarial learning framework optimizes the seed point pattern *S* defining the tessellation. In each iteration, the point pattern is adjusted based on feedback from the discriminator *D*, while *D* is simultaneously trained to improve its ability to distinguish between 2D input and tessellation-based data. The update of the seed points corresponds to a local displacement of individual seed points, which allows for the local adaptation of the tessellation in regions where the discriminator detects statistical discrepancies between the cellular input structure and planar sections of the tessellation. Optimization is driven by two loss functions $L_\textrm{T},L_\textrm{D}$: the first is used to rate the realism of a tessellation $C^S$, and thus is used to adjust the corresponding seed point pattern *S*, while the second is used to train the discriminator *D*, that is, to refine the learned statistics.

For a Voronoi tessellation $C^S$ and some corresponding RGB colors $c^S$ (see Eq. (2)) the loss $L_\textrm{T}(D,C^S,c^S)$ is given by

8 $$\begin{aligned} L_\textrm{T}(D,C^S,c^S) = \frac{1}{3L} \sum \limits _{d=1}^3\sum \limits _{k=1}^L ||D(I(C^S,c^S,G_{d,k}))||_1, \end{aligned}$$

where $||\cdot ||_1$ denotes the $L_1$-almost everywhere differentiable matrix norm, and $I(C^S,c^S,G_{d,k})\in [0,1]$ is the RGB encoding of $C^S$ on the grid $G_{d,k}$, see Eq. (2). Intuitively speaking, the loss $L_\textrm{T}(D,C^S,c^S)$ defined in Eq. (8) provides the average score of *D* among all planar sections through the tessellation $C^S$ defined by the 2D grids $G_{d,k}$ for $d\in \{1,2,3\}, k\in \{0,\ldots ,{L-1}\}$. Furthermore, for a cellular input structure *C* with values known on a 2D grid *G* and corresponding colors *c*, the loss $L_\textrm{D}(D,C,c,C^S,c^S)$ is given by

9 $$\begin{aligned} L_\textrm{D}(D,C,c,C^S,c^S) = ||D(I(C,c,G))||_1 - L_\textrm{T}(D,C^S,c^S). \end{aligned}$$

Simultaneously minimizing $L_\textrm{T}$ with respect to the set *S* of seed points and $L_\textrm{D}$ with respect to the parameters of *D* within the adversarial training loop progressively improves the quality of the microstructures generated. This improvement is achieved by locally adjusting the seed points by the gradient vector of $L_\textrm{T}$ so that the resulting tessellation more accurately reproduces the local statistical features of the input data as assessed by *D*.

*Training procedure* Training starts with a set $S=\{s_1,\ldots ,s_n\}$ of independently and uniformly distributed seed points within the observation window *W*. Then the training process alternates between updating the positions of the seed points in *S* and training the discriminator *D* by means of a gradient descent-based minimization of the respective loss functions $L_\textrm{T}$ and $L_\textrm{D}$ introduced in Eqs. (8) and (9), respectively. The discriminator network *D* and the set *S* of seed points are trained using an Adam optimizer^77^ with learning rates $1 \times 10^{-5}$ and $3 \times 10^{-1}$, respectively. The framework was trained for 10,000 iterations. By randomly choosing the colors $c_i, c_i^S\in [0,1]^3$, uniformly for each cell at each optimization step for both *C* and $C^S$, implicit data augmentation is applied, mitigating the overfitting of the discriminator. This allows the pipeline to be applied to even small measured images without the risk of a so-called model collapse^75^.

Note that until now, a fixed number $n\in \mathbb {N}$ of seed points in *S* is assumed, which may not be feasible in application, since estimating the number of cells in a 3D volume based on 2D planar sections is not straightforward. To address this issue, a procedure is applied to adapt the number *n* of seed points. The procedure starts with 300 seed points and then, after every 100 iteration of the optimization loop, the average numbers $n_\textrm{2D}$ and $n'_\textrm{2D}$ of cells per unit area observed in the simulated and input 2D images, respectively, are compared. If $n_\textrm{2D} < n'_\textrm{2D}$, then $\lfloor \frac{L^3(n'_\textrm{2D}-n_\textrm{2D})}{2} \rfloor$ new seed points are added independently and uniformly distributed at random positions in the observation window $W=[0,L]^3$, and if $n_\textrm{2D} > n'_\textrm{2D}$, then $\lfloor \frac{ L^3(n_\textrm{2D}-n'_\textrm{2D})}{2} \rfloor$ existing seed points are removed at random, where $\lfloor \cdot \rfloor$ denotes rounding to the next smaller integer. This adaptive procedure allows the framework to dynamically adjust the number of seed points. Intuitively, $L^3(n'_\textrm{2D}-n_\textrm{2D})$ estimates the number of cells missing in the tessellation. By adding or removing half of this amount, one achieves a fast convergence to the correct number of seed points without overshooting.

**Fig. 3:**
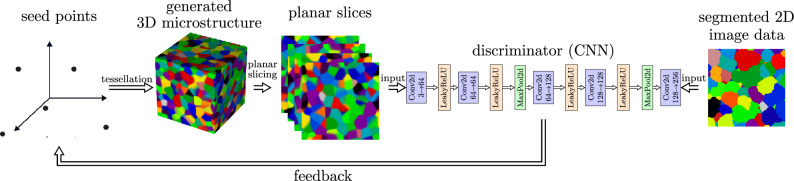
Training procedure diagram showing the adversarial optimization process.

A schematic representation of the training procedure is shown in Fig. 3, where the discriminator obtains segmented 2D image data and planar sections of generated 3D microstructures, subsequently optimizing the seed points that generate the 3D microstructure by a Voronoi tessellation.

In the present work, a single discriminator evaluates the quality of 2D planar tessellation sections in all directions $d\in \{1,2,3\}$, restricting the framework to generate so-called isotropic cellular structures, that is, cellular structures whose statistics do not change under rotation of the microstructure. However, the framework can be directly extended to generate tessellations with preferred cell orientations by employing multiple discriminators with identical architectures but independent parameters, each trained for unique direction-specific statistics^39^.

## Results and discussion

We now provide a detailed evaluation of the potential of the stereological framework presented in section “Stereological framework for generating 3D microstructures” to generate 3D cellular structures. Therefore, we will introduce metrics that will quantify the discrepancies between generated and input structures. Note that such structural discrepancies can have various causes, e.g. (1) the presented encoding and GAN-based optimization procedure is not suitable for the optimization of point patterns and corresponding tessellations, (2) the 2D data may not contain sufficient information to fully reconstruct the corresponding 3D structure (unrepresentative data) or the model may not be able to solve the stereological problem, and (3) the use of Voronoi tessellations may impose restrictive assumptions that limit the representational capacity of the framework. Each of these three issues is systematically examined in the following to evaluate the applicability of the proposed framework.

### Geometric descriptors for microstructure characterization

The evaluation of the stereological framework presented in section “Stereological framework for generating 3D microstructures” is performed by comparing the empirical distributions of geometric cell descriptors in the reconstructed microstructures with those of the input structures. The descriptors considered include the volume, surface area, and elongation of cells, as well as the number of neighboring cells. In the following, these descriptors are introduced for cells observed in both 2D and 3D images.

Let *G* denote a grid of pixels (2D) or voxels (3D) of an image on which some cellular structure $C=(C_1,\ldots ,C_n)$ is defined; see section “Cellular structure and RGB representation”. Then, for each $i\in \{1,\ldots ,n\}$, the volume $V(C_i)$ of the *i*th cell $C_i$ is given as the number of pixels/voxels belonging to this cell, i.e. the number of pixels/voxels $x\in G$ for which $C_i(x)=1$ holds. The area/volume equivalent diameter $d_{V}(C_i)$ is given by the diameter of a sphere with the same volume as $C_i$, i.e.

10 $$\begin{aligned} d_{V}(C_i) = 2 \left( \frac{V(C_i)}{\pi } \right) ^{1/2} \quad \text {in 2D},\quad \hbox {and}\quad d_{V}(C_i) = 2 \left( \frac{3 V(C_i)}{4 \pi } \right) ^{1/3} \quad \text {in 3D}. \end{aligned}$$

The surface area $S_A(C_i)$ of a cell $C_i$, often referred to as the perimeter in 2D, is computed using a marching cube algorithm implemented in Ref.^78^ for 2D images and the method described in Ref.^79^ for 3D images. These approaches try to mitigate common discretization artifacts in surface area estimation arising from pixel or voxel representations. The elongation $E(C_i)$ of a cell $C_i$ is defined as the ratio of the smallest to the largest eigenvalue of the principal component analysis of all $x\in G$ for which $C_i(x)=1$ holds, applied in 2D or 3D, that is,

11 $$\begin{aligned} E(C_i) = \frac{\lambda _{\min }(C_i)}{\lambda _{\max }(C_i)}, \end{aligned}$$

where $\lambda _{\max }(C_i),\lambda _{\min }(C_i)\ge 0$ denote the largest and smallest eigenvalues, respectively. The number of neighbors $\gamma (C_i)$ of a cell $C_i$ is defined as the number of distinct cells $C_j \subset G$, $j \ne i$, such that $C_j \in G$ shares at least one pixel/voxel within the 8-neighborhood (in 2D) or the 26-neighborhood (in 3D) of any pixel/voxel in $C_i$. In the following, a 2D (or 3D) descriptor denotes a descriptor computed on a 2D (or 3D) image, while the descriptor itself always takes a scalar value.

### Validation on synthetic microstructures

To investigate issues (1) and (2) stated above, synthetic 3D microstructures based on Voronoi tessellations are generated as input data. This makes it possible to evaluate the performance of the stereological reconstruction procedure while excluding the possibility that issue (3) occurs, namely, that a Voronoi tessellation model is inadequate for representing the input data.

*Generation of seed points* To produce a variety of synthetic cellular structures, three distinct point patterns are generated within the observation window $W = [0,100]^3$ with periodic boundary conditions. Voronoi tessellations are then constructed on the basis of these point patterns. Each of the generated point patterns consists of 666 points allowing for their easy comparison, and the corresponding 3D Voronoi tessellations represent microstructures commonly observed in practical applications. In the first of the three cases, seed points are sampled independently and uniformly distributed in *W*, representing the most simple way to generate cellular structures^80^. The resulting point pattern is from now on called a uniform point pattern. In the second case, seed points are uniformly sampled in *W* conditioned on a minimum distance of 10 voxels between two points, producing a microstructure with a controlled minimal cell size^81^. The resulting point pattern is called a hardcore point pattern. In the third case, 222 points are uniformly sampled in *W*, and for each of these initial points, a randomly oriented straight line is generated, which passes through this point. Two points are placed on each line at a distance of 10 voxels from the initial point, so that the initial point lies between these two newly placed points. The tessellation generated from this point pattern exhibits a clustering behavior of cells similar to that observed in strongly twinned metallic alloys^82^. This point pattern is called a twinning point pattern.

**Fig. 4:**
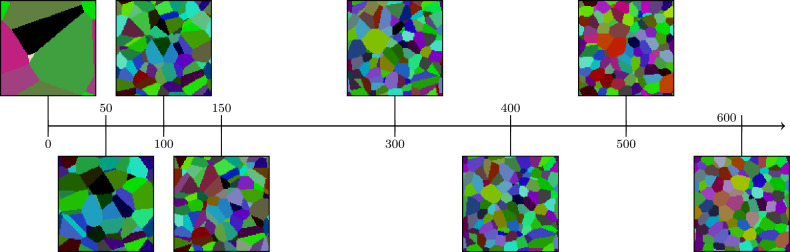
A planar section of the microstructure during the generation of the 3D microstructure in case of the hardcore point pattern. The horizontal arrow indicates the corresponding training iteration.

**Fig. 5:**
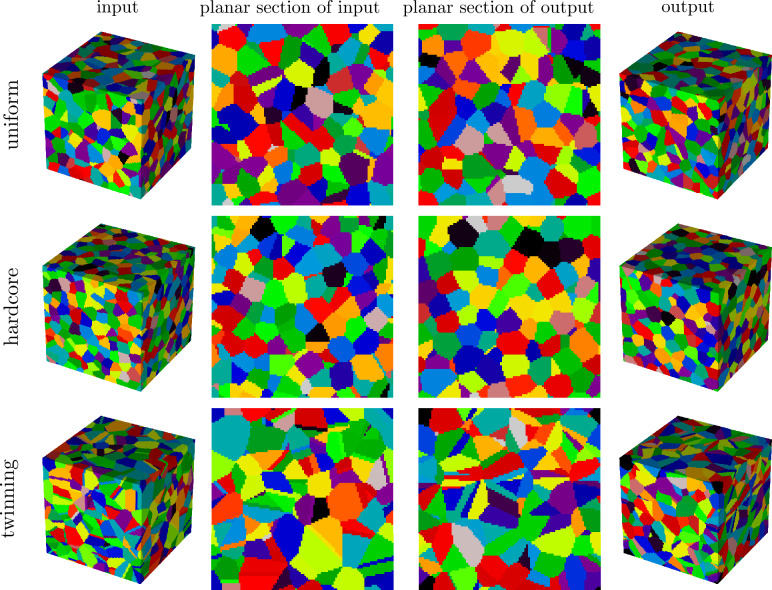
Discretized Voronoi tessellations generated by uniform, hardcore, and twinning point patterns (from top to bottom), used as input structures, are shown in the first column. The stereologically generated 3D microstructures (output) are shown in the fourth column. Planar sections of input and output structures for the different point patterns are visualized in the second and third column.

*Construction of synthetic microstructures* Voronoi tessellations are constructed from the three point patterns stated above, following the procedure outlined in section  "Voronoi tessellation”, and subsequently evaluated on the 3D voxel grid given by $W\cap \{0,1,\ldots \}^3$ to obtain 3D image representations. These images serve as synthetic microstructures for evaluating the performance of the stereological reconstruction framework. For a visualization of the point patterns and corresponding tessellations, see the Appendix. Furthermore, Fig. 4 shows the evolution of the tessellation during microstructure generation and Fig. 5 a visualization of the three different microstructures.

*Comparison of descriptor distributions*

**Fig. 6:**
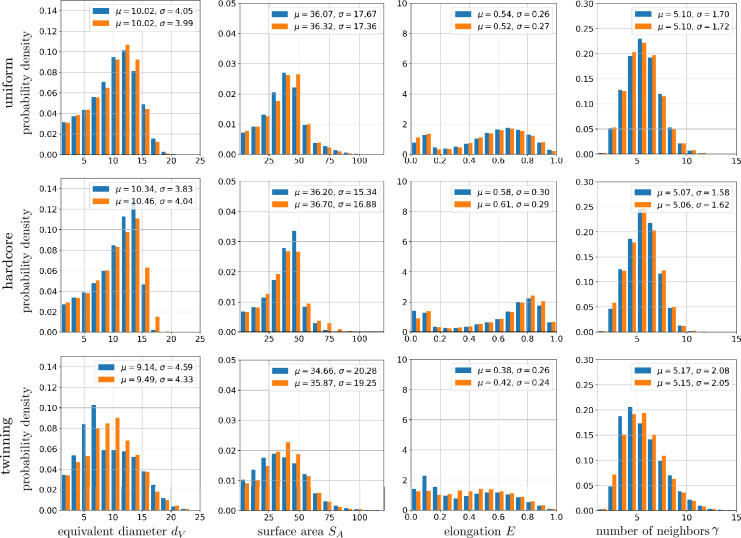
Histograms of geometric 2D descriptors together with their mean ($\mu$) and standard deviation ($\sigma$) values computed from planar sections of input (blue) and output (orange) structures obtained from the stereological reconstruction framework. The input structures are generated by Voronoi tessellations based on uniform, hardcore and twinning point patterns (from top to bottom).

To prepare the synthetic microstructures as input for the stereological reconstruction procedure, 2D image data is required, as described in section “Stereological framework for generating 3D microstructures”. Thus, for each synthetic 3D structure, planar 2D sections are extracted along the three principal coordinate directions, resulting in three hundred 2D images per microstructure. These images serve as training input for the stereological reconstruction framework per synthetic structure, where the synthetic 3D microstructures are referred to as input, and the resulting 3D structures generated by the stereological reconstruction method are called output structures. The fourth column of Fig. 5 displays the output for the three inputs. Visual comparison reveals good agreement between the input and the corresponding output. Thus, the framework visually captures not only the uniform case, but also the almost regular structure in the hardcore case. Furthermore, in the case of twinning, more elongated cells with twinning-like features, i.e. cells with flat boundaries, are visible in the input structure as well as in the corresponding planar section of the output structure, see Fig. 5. To investigate possible discrepancies between input and output that may have been caused by issue (1), planar sections of the input and output structures are statistically compared using the geometric descriptors introduced in section “Geometric descriptors for microstructure characterization”. Note that, since both input and output structures are based on periodic Voronoi tessellations, the periodic boundary conditions are taken into account in the computation of the descriptors. It can be observed that an extremely high agreement is achieved between the histograms of the considered descriptors; see Fig. 6. This indicates that the framework can properly optimize seed point patterns in 3D such that 3D Voronoi tessellations with desired 2D sections are generated.

**Fig. 7:**
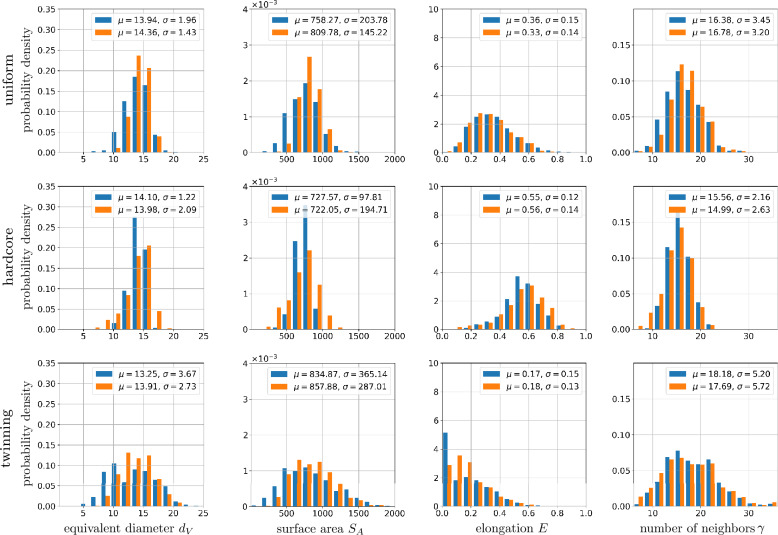
Histograms of geometric descriptors of 3D microstructures together with their mean ($\mu$) and standard deviation ($\sigma$) values computed from input (blue) and output (orange) images. The input structures are generated by Voronoi tessellations based on uniform, hardcore and twinning point patterns (from top to bottom).

To assess the influence of issue (2) on the quality of the framework output, the histograms of 3D cell descriptors of input and output structures are compared, see Fig. 7. Importantly, despite being trained on 2D image data, the proposed method generates 3D outputs whose empirical descriptor distributions match those of the input well, although not as perfectly as in the 2D case (considered in Fig. 6). Minor discrepancies remain, particularly when considering area/volume equivalent diameters and surface areas. Furthermore, the comparison between the distributions of 2D and 3D descriptors reveals systematic differences: in the 2D case, cells from the three point patterns exhibit similar elongation values, whereas the histograms of the 3D case show different elongations. Likewise, the area/volume equivalent diameters are underestimated in 2D relative to the 3D case.

### Application to measured image data

In this section, we investigate discrepancies between input and output structures that can be attributed to issue (3). Therefore, the proposed stereological reconstruction framework is applied to data originating from real-world materials. For this, 3D image data depicting the cellular structure of three distinct materials are considered to showcase the method’s versatility across different length scales and structural characteristics. More precisely, 3D image data of foams^17^, biological data of epidermal cells of a zebrafish^83^, and polycrystalline metallic materials^12^ are used to validate the framework proposed in the present paper. A more detailed description of the data sets under consideration can be found in the Appendix, in particular, how the segmented images are computed from the original raw 3D images. In the following, these datasets will be referred to as foam, biological and metallic data, respectively.

**Fig. 8:**
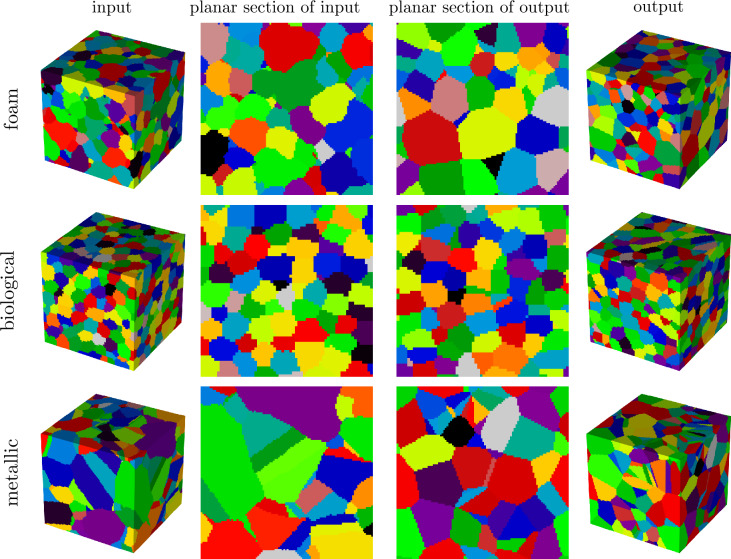
Segmented input data, computed from measured 3D images, and corresponding generated microstructures for foam (first row), biological (second row) and metallic data (third row). In the first and fourth columns the 3D input and output data are visualized, respectively, whereas in the second and third columns planar sections of corresponding data are shown.

Figure 8 shows an overview of the datasets, including segmented 3D images obtained from experimental measurements, the corresponding 3D cellular structures generated by the proposed stereological reconstruction framework, and representative planar sections of these volumes. Note that the three datasets exhibit distinct morphological characteristics: the foam data show a broad distribution of cell sizes, with smaller cells tending to cluster; the biological data exhibit an almost monodisperse distribution of nearly spherical cells, closely resembling the cell morphology in the synthetic hardcore dataset investigated in section “Validation on synthetic microstructures”; and the metallic data contain a more complex structure with elongated, parallel cells and twinning structures, similar to the synthetic twinning data set. As in the synthetic case, planar sections extracted from the 3D images serve as basis for the reconstruction framework. Visually, the reconstructed 3D cellular structures capture the variety of morphological characteristics effectively, despite the inherent constraint of representing cell boundaries with straight/planar facets.

**Fig. 9:**
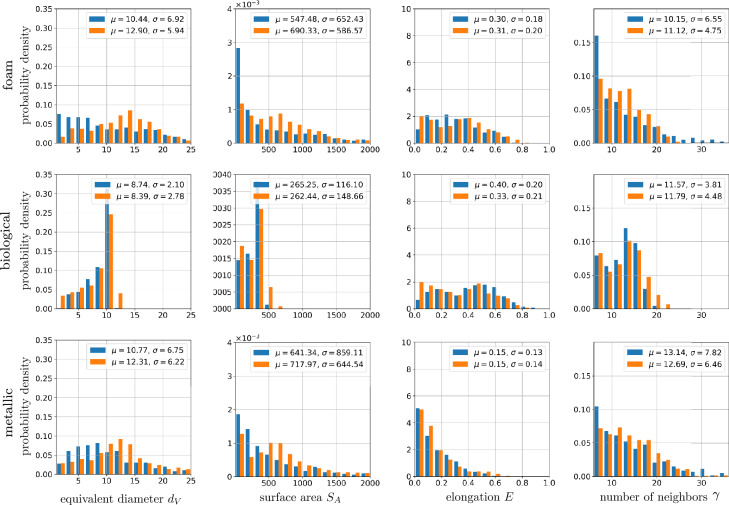
Histograms of geometric 3D descriptors, together with their mean values ($\mu$) and standard deviations ($\sigma$), for input (blue) and output structures (orange) of foam, biological and metallic data.

To quantitatively assess the differences among the three structures, geometric 3D descriptors are computed, as defined in section  “Geometric descriptors for microstructure characterization”. The histograms of these descriptors are shown in Fig. 9. They support the visual observation that cells in the biological dataset are generally smaller and exhibit less size variation compared to those in the foam and metallic data, as reflected in the empirical distributions of volume-equivalent diameter and surface area. Additionally, the characteristic twinning structure observed in the metallic data is captured by the elongation distribution, which indicates a lower number of elongated cells relative to the other two datasets.

The quantitative comparison between the measured data and the output of the reconstruction framework indicates that the geometric descriptor distributions for the output structures match the distributions obtained for the input data quite well for the three datasets. Some deviations are visible in the distributions of the volume-equivalent diameters for the three datasets because the sizes of the generated cells in the foam and metallic data are smaller than in the input structures. The same effect can be seen for the surface area. However, the elongation and number of neighbor histograms match quite well, indicating that the reconstruction framework is able to adequately generate 3D microstructures, although the generation process is restricted to Voronoi tessellations.

### Benchmarking

To the best knowledge of the authors, there is no open-source method referred to in the literature that is directly comparable to the method proposed in the present paper, in terms of stereological generation of low-parametric 3D cellular structures. Thus, our method is compared to a more general method, not generating low-parametric representations, but voxelized 3D grayscale structures. More precisely, we compare our method with SliceGAN^39^, an open-source framework based on a GAN that generates voxelized 3D structures by learning from 2D sections.

**Fig. 10:**
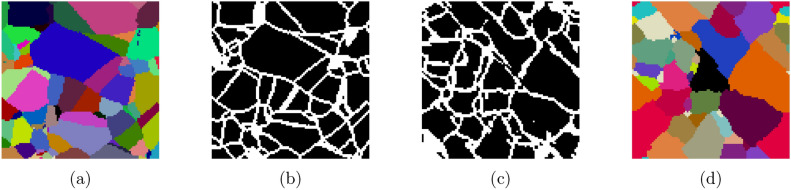
Application of SliceGAN to cellular structures. (**a**) Cell-wise colored planar section of the metallic data. (**b**) Corresponding binary image of cell boundaries. (**c**) Predicted cell boundaries produced by SliceGAN. The corresponding binary 3D image shows in total 28 disconnected (black) regions, the largest taking more than 99% of the voxels. (**d**) Corresponding cell-wise segmentation and colored planar section generated by the approach described in the Appendix.

SliceGAN uses a convolutional generator network to create 3D grayscale images whose planar 2D sections are statistically similar to its training data. For applying SliceGAN to cellular data, a feasible representation of these data is required, e.g. a binary image depicting the cell boundary network. For this purpose, a 3D ground truth image is generated for each of the three real-world cellular structures considered in section “Application to measured image data”. This image assigns to each voxel the value 1 (white) if in its 6-neighborhood at least one voxel is assigned to a different cell, and the value of 0 (black) otherwise, as shown in Fig. 10a,b. Then, planar 2D sections of these binary 3D images are used to train the SliceGAN framework. However, note that the binarization procedure described above has the disadvantage that it may reduce the size of cells, in particular, leading to significant size reduction or vanishing of small cells.

An example of a planar 2D section of the 3D image generated by SliceGAN is shown in Fig. 10c. It can be observed that the cell boundaries are not always closed; thus a non-trivial post-processing step is needed to properly convert this grayscale encoding back to a cell-wise representation. For this, the procedure described in the Appendix is applied. The result of the post-processing step is visualized in Fig. 10d. It can be observed that cells generated by the SliceGAN framework do not have to be convex.

Table 1 compares the numbers of cells of the input structures with those of the corresponding reconstructions. Furthermore, Table 2 presents the relative deviations between the mean values of the geometric descriptors introduced in section “Geometric descriptors for microstructure characterization”, computed for the input structures and the generated cellular structures, respectively. In addition, the empirical Wasserstein distance^84^ between the corresponding empirical distributions of the geometric descriptors is considered, where the Wasserstein distance $\Delta (x,x')$ between two samples $x=(x_1,\ldots ,x_n)\in \mathbb {R}^n$ and $x'=(x'_1,\ldots ,x'_n)\in \mathbb {R}^{n'}$ of size $n,n'>0$ with empirical distribution functions $F_x:\mathbb {R}\rightarrow [0,1]$ and $F_{x'}:\mathbb {R}\rightarrow [0,1]$ is defined as

12 $$\begin{aligned} \Delta (x, x') = \int _\mathbb {R}|F_x(t)-F_{x'}(t)| \,\textrm{d}t\,. \end{aligned}$$

The value $\Delta (x,x')$ has the physical dimension of the underlying descriptor and can be interpreted as the average amount by which the descriptor values must be shifted to transform one empirical distribution function into the other, i.e. larger values correspond to larger systematic differences in characteristic cell sizes, etc.

Overall, both methods perform reasonably well with respect to stereological reconstruction of all three real-world datasets introduced in section “Application to measured image data”. However, the method proposed in the present paper shows a clear advantage in predicting the correct number of cells; see Table 1. This probably stems from the presence of irregular or thin cells in the datasets, which are challenging to reconstruct with the SliceGAN method on the basis of the chosen encoding approach, i.e. using binary images of cell boundary networks. Furthermore, the framework proposed in the present paper tends to produce more accurate reconstructions with respect to the volume-equivalent diameter and the surface area of cells. However, SliceGAN performs better in terms of reconstructing cell elongation and the number of cell neighbors. These differences can be attributed to the more flexible voxel-based representation of cellular structures when using SliceGAN, which allows for arbitrary cell shapes but requires complex post-processing to identify individual cells. Note that this post-processing may introduce additional errors; see the Appendix for a more detailed comparison. Overall, the comparison suggests the following practical guideline. If a low-parametric, cell-wise, and physically interpretable 3D representation is required (e.g. for parameter studies, statistical analysis, or direct mesh generation), the tessellation-based approach is preferable. If accurately capturing highly non-convex morphologies or strongly curved boundaries is the primary objective, voxel-based methods can be advantageous, but typically require non-trivial postprocessing to recover a valid cell segmentation.

**Table 1:** Total number of cells in input and output structures for both reconstruction methods, along with the relative error percentages.

Structure	Ground truth	Voronoi tessellation	SliceGAN
Foam	672	534	457
Biological	647	655	749
Metallic	715	554	416

**Table 2:** Relative deviations between mean descriptor values of input and generated microstructures (in %) and empirical Wasserstein distance computed for input structures and generated cellular structures.

Structure	Descriptor	Method	Mean error %	Wasserstein distance
Foam	Equivalent diameter $d_{V}$	Tessellation	23.6	2.507
		SliceGAN	27.9	2.944
	Surface area $S_A$	Tessellation	26.1	167.729
		SliceGAN	44.5	245.221
	Elongation $E$	Tessellation	3.1	0.029
		SliceGAN	2.1	0.013
	Number of neighbors $\gamma$	Tessellation	9.5	2.095
		SliceGAN	0.8	1.052
Biological	Equivalent diameter $d_{V}$	Tessellation	4.1	0.623
		SliceGAN	39.3	3.771
	Surface area $S_A$	Tessellation	1.4	30.324
		SliceGAN	122.9	333.488
	Elongation $E$	Tessellation	18.3	0.073
		SliceGAN	12.5	0.050
	Number of neighbors $\gamma$	Tessellation	1.5	0.554
		SliceGAN	5.1	0.942
Metallic	Equivalent diameter $d_{V}$	Tessellation	21.5	2.374
		SliceGAN	23.6	2.451
	Surface area $S_A$	Tessellation	20.9	207.644
		SliceGAN	39.6	236.791
	Elongation $E$	Tessellation	10.8	0.016
		SliceGAN	88.1	0.120
	Number of neighbors $\gamma$	Tessellation	2.9	1.207
		SliceGAN	15.2	2.004

High values indicate large dissimilarities between input and output structures.

### Discussion on model selection

As indicated in sections “Validation on synthetic microstructures” and “Application to measured image data”, the proposed method is generally capable of generating cellular structures that resemble both virtually generated and experimentally measured datasets exhibiting various microstructural morphologies. However, generating 3D microstructures based on Voronoi tessellations combined with a GAN approach imposes certain benefits and model limitations.

Voronoi tessellations inherently produce convex polyhedral cells with planar bounding facets, thus it is not possible to generate cells with curved boundaries. This limitation becomes evident for the foam data, where the experimental cells exhibit more rounded geometries. Although the volume-equivalent diameters of the generated cells follow the experimental size distribution reasonably well, the distributions of cell surfaces show poorer visual agreement. A further limitation, also linked to the choice of the tessellation model, concerns datasets with multimodal cell size distributions. For instance, in the metallic data, which contains both very large and small cells, the proposed framework tends to produce cells with a more homogeneous size distribution. However, these problems can be mitigated by employing more general tessellation models^40,85^. For example, multiplicatively and additively weighted Laguerre tessellations incorporate scalar weights to the seed points, which enable the generation of cells with curved boundaries and/or multimodal size distributions, thereby improving the structural similarity of the reconstruction.

Furthermore, the current implementation of the proposed framework is limited to generating isotropic tessellations, as it employs a single discriminator for all spatial directions. Overcoming this limitation can be particularly relevant when modeling polycrystalline microstructures, where cells can exhibit preferred orientations relative to specific crystallographic or processing directions. Extending the framework to anisotropic structures would require the training of multiple discriminators, each on planar sections aligned with different orientations. However, for applications involving real-world data, this extension necessitates prior knowledge of the relative orientations between the available 2D input sections in order to allow for similarly oriented virtual planar sectioning during the framework training.

The stereological reconstruction approach based on tessellations offers a low-parametric representation of cellular 3D microstructures compared to alternative frameworks such as SliceGAN. With this parametric representation, a reduction of dimensionality of the parameter space by a factor of about 500 was achieved. Furthermore, the output of the proposed method inherently provides cell-wise segmentation, which other methods do not guarantee without additional nontrivial post-processing steps as seen in section “Benchmarking”. Moreover, the continuous representation obtained from the tessellation can be directly converted into surface meshes or other representations required by subsequent simulation tools, e.g. finite element simulations. This eliminates the need for complex mesh extraction and smoothing steps often required for voxelized datasets^62,86^.

## Conclusion

This paper presents a novel stereological framework that combines differentiable Voronoi tessellations with adversarial learning to generate cellular 3D microstructures from two-dimensional image data. The method addresses a critical challenge in materials science by providing a computationally efficient and physically interpretable approach to reconstructing 3D microstructures without the need for expensive or destructive 3D imaging techniques.

The key innovation lies in integrating a differentiable Voronoi tessellation with a patch-based discriminator network, enabling gradient-based optimization of seed point patterns of Voronoi tessellations to match statistical properties of measured planar 2D sections. This approach generates low-parametric cellular 3D structures that are resolution-independent and computationally efficient.

Validation for synthetic and real datasets demonstrates effectiveness in preserving essential geometric and statistical properties of various material systems, including foams, biological tissues, and metallic alloys. Although the current implementation is restricted to the generation of cellular structures with convex polyhedral cells, the framework can nevertheless be applied to more general microstructures, still capturing the essential features of the input data.

## Supplementary Information

Supplementary Information.

## Data Availability

The data is available from the authors upon reasonable request.
