# Supplementary material for: Stereological reconstructions of 3D cellular microstructures by combining adversarial learning and Voronoi tessellations
Source: Sci Rep. 2026 May 13;16:15058. doi: 10.1038/s41598-026-52851-7 (PMC13172447; doi:10.1038/s41598-026-52851-7)
Supplement: Supplementary file 1 — Supplementary Information. [file 41598_2026_52851_MOESM1_ESM.pdf]

Appendix

A Neural network architecture

Table 1 summarizes the architecture of the discriminator network used in this paper. The receptive field (RF) indicates the effective field of view of each layer, that is, the maximum size of a spatial region of the input image that can influence the output at a given layer. Note that the features relevant for the discriminator must lie within the receptive field of the 11th layer. Therefore, the value of L governing the resolution or size of the observation window must be chosen appropriately.

Definitions of layer types, kernel sizes, and other parameters can be found in the standard deep learning literature; see e.g. [1], as well as in the PyTorch documentation. The implementation of the network considered in the present paper is based on PyTorch [2].

Table 1: Discriminator architecture. Columns: Layer (sequential index), type (layer class), input (number of input channels), output (number of output channels), kernel (kernel size), params (number of trainable parameters), RF (receptive field size)

layer	type	input	output	kernel	params	RF	notes
1	Conv2D	3	64	3×3	1.7K	3	no bias, spectral norm
2	LeakyReLU	-	-	-	0	-	slope=0.2, inplace
3	Conv2D	64	64	3×3	36.9K	5	no bias, spectral norm
4	LeakyReLU	-	-	-	0	-	slope=0.2, inplace
5	MaxPool2D	-	-	2	0	6	-
6	Conv2D	64	128	3×3	73.7K	10	no bias, spectral norm
7	LeakyReLU	-	-	-	0	-	slope=0.2, inplace
8	Conv2D	128	128	3×3	147.5K	14	no bias, spectral norm
9	LeakyReLU	-	-	-	0	-	slope=0.2, inplace
10	MaxPool2D	-	-	2	0	16	-
11	Conv2D	128	256	3×3	294.9K	24	no bias, spectral norm

B Visualization of point patterns and corresponding tessellations

A visualization of the point patterns and the corresponding tessellations used for the generation of synthetic cellular structures is shown in Figure 1. For clarity 2D point patterns are considered and the tessellations are displayed without periodic boundary conditions.

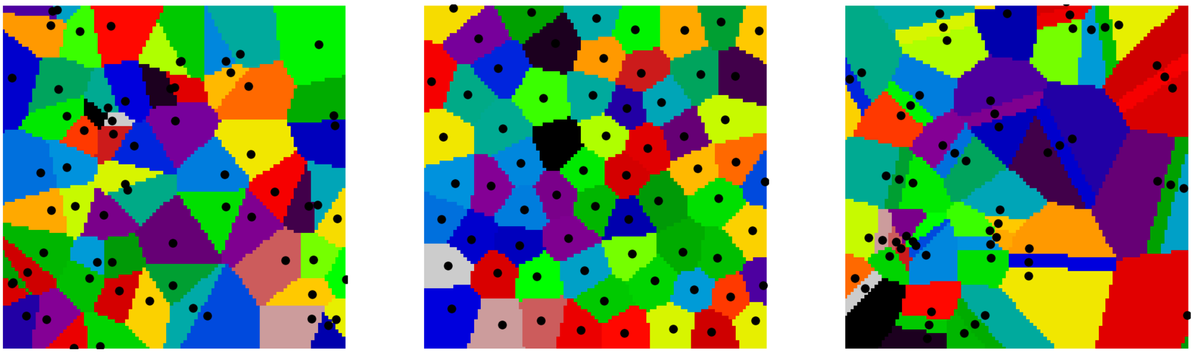

(a) uniform (b) hardcore (c) twinning

Figure 1: Visualization of point patterns. Seed points are shown in black.

C Description of measured data

This section describes the experimental datasets to which the proposed stereological reconstruction method is applied. In this study, three different datasets are considered: 3D image data of polymer foams [3], biological data of epidermal cells from a zebrafish brain [4], and polycrystalline metallic microstructures [5]. The reconstruction framework relies on a space-filling segmentation into individual cells. Consequently, for each dataset, the preprocessing steps required to achieve such a segmentation are detailed, where applicable.

C.1 Foam data

The foam database provided in [3] contains structure–property and mechanical modeling data for elastic impact protection foams, obtained from various imaging techniques (e.g. microcomputed tomography, digital image correlation) and force-sensing methods (e.g. dynamic mechanical analysis, universal testing) under diverse experimental conditions and loading modes. Among the different image data types available, the present paper employs the binarized 3D datasets (Figure 2a) with a voxel side length of 4 µm, where the background (black) corresponds to the foam and the foreground (white) to the foam cells. These binarized data do not directly provide a segmentation into individual foam cells, because the cells are partially interconnected, making it impossible to identify single cells as connected components. Therefore, the data are preprocessed by first employing a morphological dilation [6] on the original binarized data (Figure 2b), followed by a combination of the watershed algorithm [7–10] and a 3D morphological reconstruction approach [11, 12] to segment the binarized images into individual foam cells while simultaneously preventing oversegmentation (Figure 2c). Finally, the cubic image was rescaled to a resolution of 100^3 with a voxel side length of 256 µm.

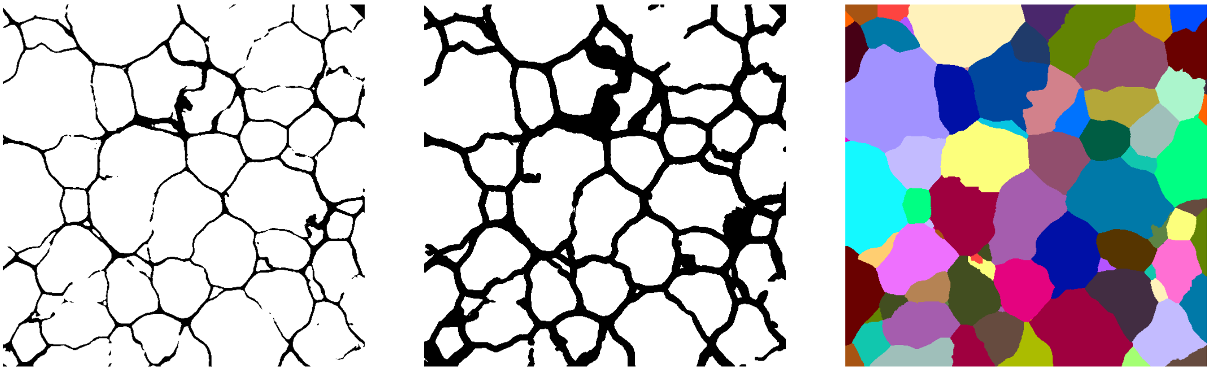

(a) (b) (c)

Figure 2: Foam data preparation. (a) Planar section of the binarized 3D image data of the foam (black) and the foam cells (white). (b) Corresponding planar section of the 3D image after applying morphological dilation and (c) space-filling segmentation achieved by means of watershed segmentation and morphological reconstruction.

C.2 Biological data

The biological dataset provided in [4] comprises 3D image data of a zebrafish brain obtained by serial-section SEM (Figure 3a), along with a corresponding segmentation of individual cells from the grayscale image (Figure 3b). The original resolution of this image data is 256 nm × 256 nm × 256 nm in x-, y-, and z-direction. However, this segmentation is not space-filling, as it includes both segmented cells and background regions. Therefore, the background voxels are reassigned to individual cells using nearest-neighbor interpolation. More precisely, each background voxel is

assigned to the nearest cell based on the minimum Euclidean distance. The resulting space-filling segmentation is shown in Figure 3c.

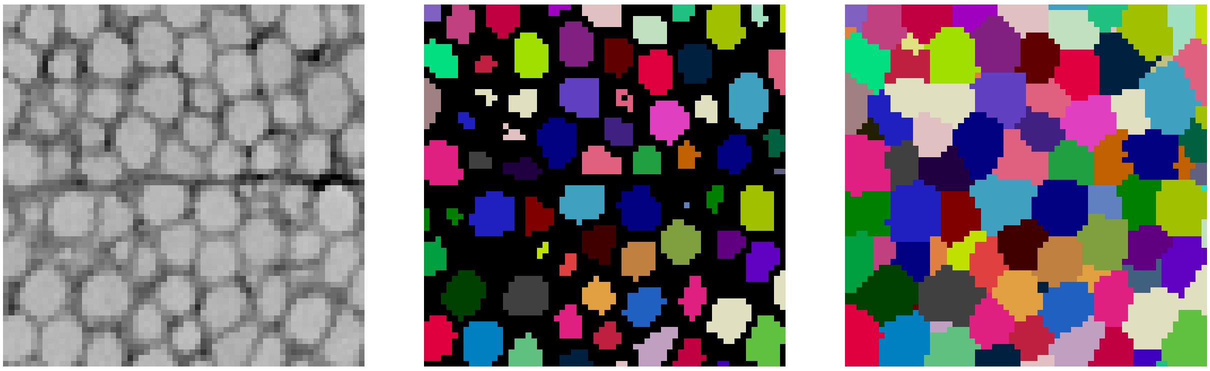

(a) (b) (c)

Figure 3: Biological data preparation. (a) Planar section of 3D SEM data of a zebrafish brain. (b) Corresponding segmented data consisting of background (black) and differently colored cells. (c) Space-filling segmentation achieved by means of nearest-neighbor interpolation.

C.3 Metallic data

The metallic dataset provided in [5] consists of 3D image data measured by electron backscatter diffraction (EBSD) of a polycrystalline metallic material. The image has a size length of 1 µm. For this data, a space-filling segmentation into individual cells is already available in [5].

D Validation results for synthetic microstructure generation

Table 2 summarizes the relative deviations between the mean values of the considered geometric descriptors evaluated for the input structures obtained from uniform, hard-core, and twinning point patterns and for the corresponding generated 3D microstructures. Moreover, it reports the empirical Wasserstein distances between the corresponding empirical distributions of the geometric descriptors.

Table 2: Relative deviations between mean descriptor values of input and generated microstructures (in %) and empirical Wasserstein distance computed for input structures and generated cellular structures. High values indicate large dissimilarities between input and output structures.

**structure**	**descriptor**	**method**	**mean Error %**	**Wasserstein**
uniform	equivalent diameter d_V	tessellation	3.0	0.527
	surface area S_A	tessellation	6.8	63.987
	elongation E	tessellation	7.1	0.025
	number of neighbors γ	tessellation	2.4	0.422
hardcore	equivalent diameter d_V	tessellation	0.8	0.647
	surface area S_A	tessellation	0.8	79.202
	elongation E	tessellation	1.7	0.021
	number of neighbors γ	tessellation	3.7	0.583
twinning	equivalent diameter d_V	tessellation	5.0	0.983
	surface area S_A	tessellation	2.8	69.773
	elongation E	tessellation	7.2	0.029
	number of neighbors γ	tessellation	2.7	0.642

E Comparison with state-of-the-art method

In Figure 4, the distributions of geometric 3D descriptors, together with their mean values (μ) and standard deviations (σ), are given for the input data and output structures obtained by the method proposed in the present paper and by SliceGAN, respectively, using Gaussian kernel density estimators. Both methods generally show good agreement with the input data.

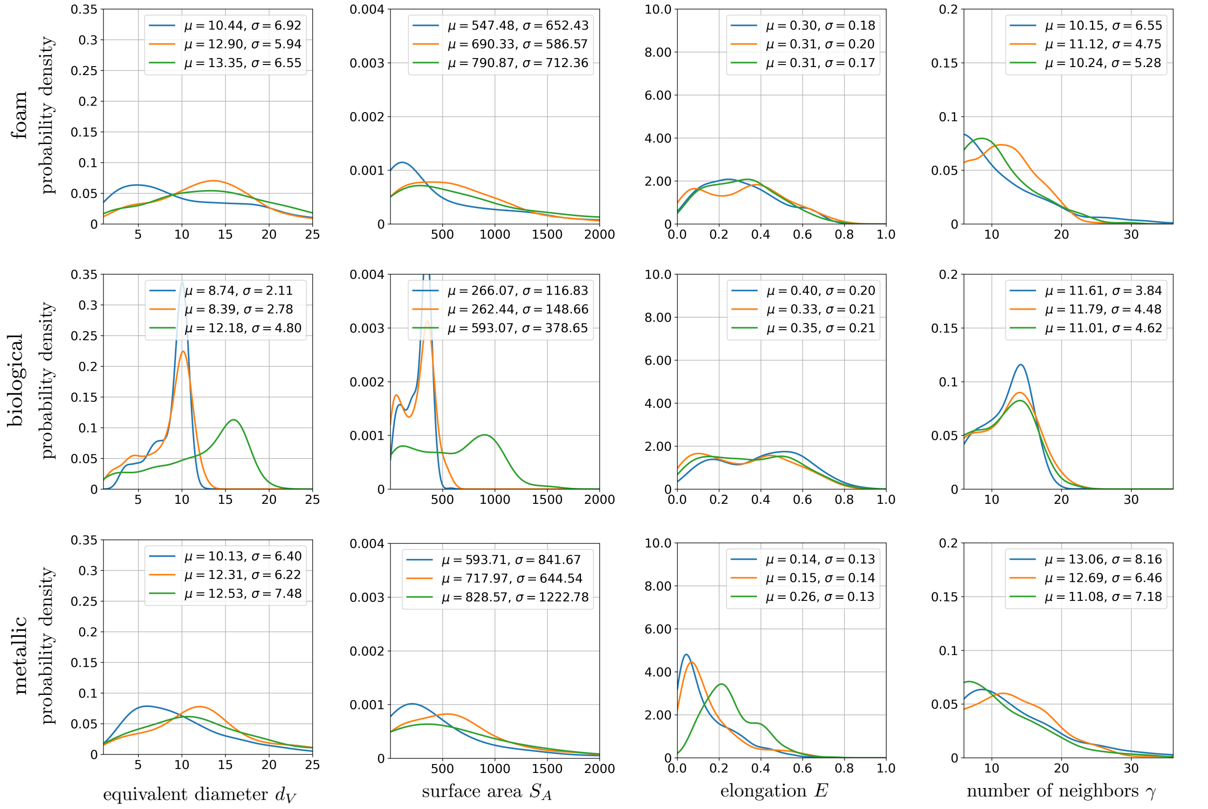

Figure 4: Distributions of geometric descriptors together with their mean values (μ) and standard deviations (σ) for input data (blue), output structures obtained by the proposed stereological reconstruction framework (orange) and the reference method (green) for foam, biological and metallic data.

References

[1] I. Goodfellow, Y. Bengio, A. Courville, and Y. Bengio. *Deep Learning*, volume 1. MIT Press, 2016.

[2] A. Paszke, S. Gross, S. Chintala, G. Chanan, E. Yang, Z. DeVito, Z. Lin, A. Desmaison, L. Antiga, and A. Lerer. Automatic differentiation in PyTorch. In *Proceedings of the 31st Conference on Neural Information Processing Systems*, pages 1–4, 2017.

[3] A.K. Landauer, O.L. Kafka, N.H. Moser, I. Foster, B. Blaiszik, and A.M. Forster. A materials data framework and dataset for elastomeric foam impact mitigating materials. *Scientific Data*, 10(1):356, 2023.

[4] Z. Lin, D. Wei, M.D Petkova, Y. Wu, Z. Ahmed, S. Zou, N. Wendt, X. Boulanger-Weill, J.and Wang, N. Dhanyasi, I. Arganda-Carreras, F. Engert, J. Lichtman, and H. Pfister. NucMM dataset: 3D neuronal nuclei instance segmentation at sub-cubic millimeter scale. In M. de Bruijne, P.C. Cattin, S. Cotin, N. Padoy, S. Speidel, Y. Zheng, and C. Essert, editors, *Medical Image Computing and Computer Assisted Intervention – MICCAI*, pages 164–174. Springer, 2021.

[5] J.-C. Stinville, J.M. Hestroffer, M.-A. Charpagne, A.T. Polonsky, M.P. Echlin, C.J. Torbet, V. Valle, K.E. Nygren, M.P. Miller, O. Klaas, A. Loghin, I.J. Beyerlein, and T.M. Pollock. Multi-modal dataset of a polycrystalline metallic material: 3D microstructure and deformation fields. *Scientific Data*, 9(1): 460, 2022.

[6] J. Serra. *Image Analysis and Mathematical Morphology.* Academic Press, 1982.

[7] S. Beucher and F. Meyer. The morphological approach to segmentation: the watershed transformation. In E.R. Dougherty, editor, *Mathematical Morphology in Image Processing*, pages 600–612. Marcel Dekker Inc., 1993.

[8] R. Beare and G. Lehmann. The watershed transform in ITK — discussion and new developments. *The Insight Journal*, 6:1–24, 2006.

[9] J.B.T.M. Roerdink and A. Meijster. The watershed transform: Definitions, algorithm and parallelization strategies. *Fundamental Informaticae*, 41(1):187–228, 2000.

[10] P. Soille. *Morphological Image Analysis: Principles and Applications.* Springer, 2013.

[11] J. Zheng and R.D. Hryciw. Segmentation of contacting soil particles in images by modified watershed analysis. *Computers and Geotechnics*, 73:142–152, 2016.

[12] A. Ridder, B. Prifling, A. Hilger, M. Osenberg, M. Weber, I. Manke, K.P. Birke, and V. Schmidt. Quantitative analysis of cyclic aging of lithium-ion batteries using synchrotron tomography and electrochemical impedance spectroscopy. *Electochemica Acta*, 444:142003, 2023.
